# Avaliação da cobertura de registro de partos no Sistema de Informação Hospitalar do Sistema Único de Saúde, segundo hospital de internação, Brasil, 2012-2020

**DOI:** 10.1590/0102-311XPT225623

**Published:** 2025-02-07

**Authors:** Juliana Alves Marques, Rosa Maria Soares Madeira Domingues, Marcos Augusto Bastos Dias, Claudia Medina Coeli, Valéria Saraceni, Rejane Sobrino Pinheiro

**Affiliations:** 1 Programa de Pós-graduação em Gestão Urbana, Pontifícia Universidade Católica do Paraná, Londrina, Brasil.; 2 Instituto Nacional de Infectologia Evandro Chagas, Fundação Oswaldo Cruz, Rio de Janeiro, Brasil.; 3 Instituto Nacional de Saúde da Mulher, da Criança e do Adolescente Fernandes Figueira, Fundação Oswaldo Cruz, Rio de Janeiro, Brasil.; 4 Instituto de Estudos em Saúde Coletiva, Universidade Federal do Rio de Janeiro, Rio de Janeiro, Brasil.; 5 Secretaria Municipal de Saúde do Rio de Janeiro, Rio de Janeiro, Brasil.

**Keywords:** Bases de Dados Estatísticos, Sistemas de Informação, Parto, Nascido Vivo, Assistência Hospitalar, Statistical Databases, Information Systems, Parturition, Live Birth, Hospital Care, Bases de Datos Estadísticos, Sistemas de Información, Parto, Nacido Vivo, Atención Hospitalaria

## Abstract

O objetivo foi avaliar a cobertura nacional de registro de partos no Sistema de Informação Hospitalar do Sistema Único de Saúde (SIH/SUS), segundo o hospital de ocorrência, e verificar características institucionais associadas. Realizou-se estudo ecológico descritivo com dados do SIH/SUS, do Sistema de Informações sobre Nascidos Vivos (SINASC) e do Cadastro Nacional de Estabelecimentos de Saúde (CNES) de acesso público, de 2012-2020. Internações de mulheres de 10 a 49 anos para parto vaginal ou cesariana no SIH/SUS foram comparadas aos registros de nascidos vivos ocorridos em partos hospitalares em estabelecimentos com convênio SUS e mais de 100 nascidos vivos/ano no SINASC. A cobertura foi medida pela proporção de internações para partos (SIH/SUS) em relação ao total de nascidos vivos SINASC. Modelos de classificação supervisionada, árvore de decisão e floresta aleatória foram utilizados para identificar as características hospitalares para a predição da cobertura. A cobertura no SIH/SUS foi estimada em 86,9%, e em 80,6% após a exclusão de hospitais com cobertura > 100%. Maiores coberturas foram observadas nas regiões Norte e Nordeste e menores no Sul e no Centro-oeste. Houve aumento da cobertura nacional de 77,9% para 82,3% no período. Os principais fatores preditivos foram a proporção de cesarianas, a quantidade de leitos obstétricos do SUS, a esfera administrativa e o porte do hospital, com menor cobertura em serviços que apresentam maior prevalência de cesarianas. Falhas no registro do CNES foram identificadas no SINASC. A cobertura de registro de partos pelo SIH/SUS é elevada, sendo menor em hospitais com elevada proporção de cesarianas. Estratégias de melhoria contínua da qualidade de registro nos sistemas de informação são necessárias.

## Abstract (en)

This study aimed to evaluate the national coverage of birth registration within the Brazilian Hospital Information System of the Brazilian Unified National Health System (SIH/SUS), according to the hospital of birth, and identify associated institutional characteristics. A descriptive ecological study was conducted using available data from the SIH/SUS, the Brazilian Information System on Live Births (SINASC), and the Brazilian National Registry of Health Establishments (CNES) from 2012 to 2020. Hospital admissions of women aged 10 to 49 for vaginal childbirth or cesarean section in the SIH/SUS were compared to live birth records from hospital deliveries in SUS-contracted establishments with more than 100 live births per year in SINASC. Coverage was measured by the proportion of admissions for childbirth (SIH/SUS) in relation to the total number of live births in SINASC. Supervised classification models, decision trees, and random forests were used to identify hospital characteristics predictive of coverage. Coverage in the SIH/SUS was estimated at 86.9%, and 80.6% after excluding hospitals with > 100% coverage. Higher coverage rates were observed in the North and Northeast regions, whereas lower rates were seen in the South and Central-West regions. National coverage increased from 77.9% to 82.3% during the period. The main predictive factors were the proportion of cesarean sections, the number of obstetric beds of SUS, the administrative sphere, and facility size, with lower coverage in services with a higher proportion of cesarean sections. Inconsistencies in CNES registration were identified in SINASC. Birth registration coverage in the SIH/SUS is high across the country, though lower in hospitals with a high proportion of cesarean sections. Strategies for continuous improvement of quality for information system records are necessary.

## Abstract (es)

El objetivo fue evaluar la cobertura nacional de registro de partos en el Sistema de Información Hospitalaria del Sistema Único de Salud brasileño (SIH/SUS), según el hospital de ocurrencia y verificar las características institucionales asociadas. Se realizó un estudio ecológico descriptivo con datos del SIH/SUS, el Sistema de Información de Nacidos Vivos (SINASC) y el Catastro Nacional de Establecimientos de Salud (CNES), de acceso público, en el período 2012-2020. Se compararon las hospitalizaciones de mujeres de 10 a 49 años para parto vaginal o cesárea en el SIH/SUS con los registros de nacidos vivos ocurridos en partos hospitalarios en establecimientos con convenio SUS y más de 100 nacidos vivos/año en el SINASC. La cobertura se midió por la proporción de hospitalizaciones por parto (SIH/SUS) con relación al total de nacidos vivos SINASC. Se utilizaron modelos de clasificación supervisada, árbol de decisión y bosque aleatorio para identificar las características hospitalarias para predecir la cobertura. La cobertura en el SIH/SUS se estimó en un 86,9% y un 80,6% después de excluir los hospitales con cobertura > 100%. Se observó mayor cobertura en las regiones Norte y Nordeste y menor en las regiones Sur y Centro-Oeste. Hubo un aumento en la cobertura nacional del 77,9% al 82,3% en este período. Los principales factores predictivos fueron la proporción de cesáreas, las camas obstétricas del SUS, el ámbito administrativo y el tamaño del hospital, con menor cobertura en los servicios con mayor proporción de cesáreas. En el SINASC se identificaron fallas en el registro del CNES. La cobertura de registro de partos en el SIH/SUS del país es alta, y es menor en hospitales con alta proporción de cesáreas. Se requieren estrategias para la mejora continua de la calidad de los registros en los sistemas de información.

## Introdução

O Sistema de Informação Hospitalar do Sistema Único de Saúde (SIH/SUS) foi implantado em 1991 e tem como principal objetivo o ressarcimento das despesas das internações ocorridas em hospitais públicos e conveniados ao SUS ^1^. Embora não seja um sistema de informação concebido para a vigilância em saúde, é utilizado em estudos de avaliação da morbidade hospitalar ^2^ ^,^ ^3^ ^,^ ^4^ ^,^ ^5^, incluindo a morbidade materna ^6^ ^,^ ^7^ ^,^ ^8^ ^,^ ^9^ ^,^ ^10^ ^,^ ^11^ ^,^ ^12^.

A avaliação da morbidade materna é recomendada pela Organização Mundial da Saúde (OMS) desde 2011, como estratégia complementar ao estudo do óbito materno ^13^ e vários estudos nacionais já utilizaram o SIH/SUS para estimar o número de casos de morbidade materna, sua variação no tempo, suas principais causas, bem como sua distribuição nas regiões do país e segundo características maternas ^6^ ^,^ ^7^ ^,^ ^9^ ^,^ ^10^ ^,^ ^11^ ^,^ ^12^.

Entretanto, para que o SIH/SUS possa ser usado para a vigilância da morbidade materna, é necessário avaliar sua validade para essa finalidade. Entre os atributos dos sistemas de informação, a cobertura avalia o grau em que os eventos de interesse estão registrados ^14^. Um único estudo nacional estimou a cobertura do registro de partos no SIH/SUS em 2009, utilizando o Sistema de Informações sobre Nascidos Vivos (SINASC) como referência ^15^. No Rio de Janeiro, dois estudos fizeram essa avaliação: um utilizando como referência um estudo de base hospitalar realizado nos anos 1999-2001 ^16^, e outro utilizando o SINASC como referência no período de 2014-2016 ^12^. Não foram identificados estudos de abrangência nacional que tenham estimado a cobertura de registro de partos no SIH/SUS após 2010.

A implantação de um sistema de vigilância da morbidade materna no Brasil é essencial para subsidiar as estratégias de melhoria do cuidado obstétrico no país, visando à redução da mortalidade materna ^17^. Considerando que o SIH/SUS é o único sistema de informação que dispõe de dados de morbidade em internações obstétricas, a importância de sua avaliação e seu aprimoramento constante e a ausência de estudos nacionais recentes avaliando a qualidade de registro nesse sistema, este artigo tem por objetivos: (1) avaliar a cobertura nacional de registro de partos no SIH/SUS, segundo o estabelecimento de ocorrência da internação; e (2) verificar as características institucionais associadas à essa cobertura, que possam orientar estratégias de melhoria da qualidade do registro.

## Métodos

### Desenho do estudo

Estudo ecológico descritivo utilizando dados nacionais do SIH/SUS, do SINASC e do Cadastro Nacional de Estabelecimentos de Saúde (CNES) disponíveis publicamente, no período 2012-2020.

### Fonte e processamento de dados

Os dados do SINASC e do SIH/SUS foram captados pelo pacote *microdatasus* por meio da linguagem de programação estatística R (https://github.com/rfsaldanha/microdatasus) ^18^ e os dados CNES foram obtidos por meio de download dos arquivos da Plataforma de Ciência de Dados Aplicadas à Saúde (PCDaS/FIOCRUZ; https://pcdas.icict.fiocruz.br/).

No SINASC, foram selecionados todos os registros de nascidos vivos, com qualquer peso ou idade gestacional, de mães de 10 a 49 anos, ocorridos em hospitais públicos ou privados conveniados ao SUS com mais de 100 nascidos vivos/ano no período de 2012-2020. Para a identificação desses hospitais, foram utilizados os dados do CNES, sendo selecionados todos os hospitais públicos e privados que dispunham de leitos obstétricos conveniados ao SUS.

No SIH/SUS, foram selecionadas todas as Autorizações de Internação Hospitalar (AIH) emitidas no período 2012-2020, de mulheres de 10 a 49 anos, com registro de procedimentos relacionados ao parto: 0310010012 (assistência ao parto sem distocia), 0310010039 (parto normal), 0310010047 (parto normal em gestação de alto risco), 0310010055 (parto normal em centro de parto normal), 0411010026 (parto cesariano em gestação de alto risco), 0411010034 (parto cesariano) e 0411010042 (parto cesariano com laqueadura tubária).

Estabelecimentos com menos de 100 nascimentos/ano, conforme registro no SINASC, foram excluídos do SIH/SUS e do SINASC, sendo essa exclusão decorrente do baixo número de nascimentos nesses estabelecimentos, o que dificultou a análise estatística, tornando-a menos robusta. Além disso, serviços com menos de 100 partos ao ano apresentam em média dois partos por semana, sendo provavelmente hospitais pequenos sem serviço estruturado de obstetrícia ou que realizam internações em obstetrícia eventualmente. Declarações de nascido vivo (DN) e AIH com número de CNES não preenchido ou com número inválido também foram excluídos da análise, já que o número do CNES foi a variável utilizada como chave identificadora nas duas bases.

No CNES, foram identificados todos os estabelecimentos com leitos obstétricos no período de 2012-2020, sendo preparada uma base anual para cada estabelecimento, contendo: número do CNES; esfera administrativa (variável “Esfera_A”, categorias municipal, estadual, federal, privada, sem informação); número de leitos obstétricos (total e conveniados ao SUS); complexidade do hospital (variável “NIV_HIER”, sendo a categoria 5 de baixa complexidade; 6 e 7 de média complexidade; e 8 de alta complexidade); atividade de ensino (variável “ATIVIDAD”, sendo categoria 1 hospital universitário; 2 e 3 outras modalidades de ensino; 4 sem atividade de ensino; e 99 não informado). Como a base do CNES é mensal, para a construção da base anual, foi utilizada a média, para as variáveis quantitativas, e a moda, para as variáveis qualitativas. O mesmo critério foi utilizado para as análises para o período de 2012-2020. Estabelecimentos que durante todo o período não apresentaram leitos obstétricos conveniados ao SUS foram excluídos da análise. A escolha das variáveis do CNES foi baseada em avaliação por especialistas.

### Cobertura de parto nos SIH/SUS

A primeira etapa da análise foi o cálculo da cobertura de registro de partos no SIH/SUS por estabelecimento, sendo o numerador o número de AIH com procedimentos de parto no estabelecimento no ano, dividido pelo número de nascidos vivos registrados no SINASC no mesmo estabelecimento e ano, multiplicado por 100. A identificação dos registros nas duas bases utilizou como chave o código do CNES e o ano.

**Figura 1:**
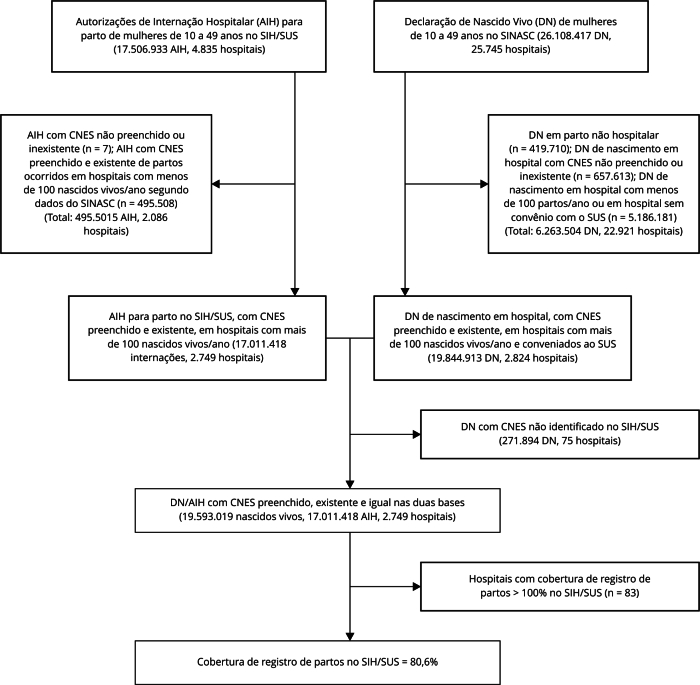
Fluxograma da análise da cobertura de partos no Sistema de Informação Hospitalar do Sistema Único de Saúde (SIH/SUS). Brasil, 2012-2020.

CNES: Cadastro Nacional de Estabelecimentos de Saúde; SINASC: Sistema de Informações sobre Nascidos Vivos.

Posteriormente, os estabelecimentos foram agrupados por Unidade Federativa (UF), sendo calculada a cobertura por UF, por macrorregião do país e país para o período 2012-2020. Foi calculada a cobertura total, considerando todos os registros, e a cobertura após exclusão dos hospitais com registro de partos no SIH/SUS superior a 100% (3% dos hospitais, 83/2.749, Figura 1). Essa exclusão se baseou na premissa de que num contexto de elevada cobertura do SINASC, uma cobertura superior a 100% no SIH/SUS seria provavelmente decorrente de erro, o que resultaria numa cobertura superestimada de registro de partos nesse sistema. A comparação da cobertura de registros de parto por macrorregião e para o país, após a exclusão dos hospitais com cobertura superior a 100%, foi avaliada considerando a tendência temporal no período de 2010-2020, sendo utilizado o teste de correlação de Spearman ^19^.

### Fatores preditores da cobertura de partos

Inicialmente, foi feita uma análise exploratória para verificar a cobertura de registro de partos no SIH/SUS segundo características hospitalares: (1) esfera administrativa (municipal, estadual, federal, privado, sem informação); (2) proporção de leitos obstétricos conveniados ao SUS, dada pela relação entre os leitos obstétricos conveniados ao SUS e o total de leitos obstétricos da unidade hospitalar, categorizada em quartis; (3) porte do hospital, considerando o número de nascidos vivos/dia registrado no SINASC (até 1, 2 a 6, ≥ 7); (4) proporção de nascimentos por cesariana, segundo dados do SINASC, categorizada em quartis; (5) complexidade do hospital (baixa, média, alta); e (6) atividade de ensino (universitário, não universitário, sem atividade de ensino).

Foram utilizados modelos de classificação supervisionados - árvore de decisão e floresta aleatória - para identificar os fatores mais relevantes para a predição da cobertura de registro de partos. Para os modelos, foram utilizados dados de treinamento na proporção de 60/40, ou seja, 60% para treinamento e 40% para teste. Foram utilizadas as classificações “cobertura adequada” e “cobertura inadequada”. Foi utilizada como parâmetro de adequação a cobertura de registro igual ou superior a 90%, valor utilizado pelo Ministério da Saúde brasileiro como parâmetro para a cobertura de registro em outros sistemas de informação no âmbito do Programa de Qualificação das Ações de Vigilância em Saúde ^20^. Foram analisadas 9.047 observações, sendo 34,2% dos hospitais considerados com cobertura inadequada. O algoritmo da árvore de decisão foi utilizado sem limitação de parâmetros para a definição da profundidade da árvore ou do número de observações nos nós. O modelo a partir do algoritmo de floresta aleatória (algoritmo de aprendizado de máquina que cria e combina árvores de decisão para obter uma única saída) foi utilizado para identificar a ordem de importância das variáveis para a classificação do perfil de cobertura dos hospitais. O algoritmo foi implementado utilizando os parâmetros para a classificação com 500 árvores e duas variáveis para a geração das árvores. A taxa de erro *out-of-bag* (OOB) estimada foi de 27,6%, indicando a porcentagem média de observações de treinamento que o modelo não classificou corretamente. Foi utilizada a métrica de *mean decrease* Gini, que avalia a importância das variáveis desse modelo (calculado com base na redução média da impureza de Gini que cada variável contribui ao longo de todas as árvores da floresta). A impureza de Gini é uma medida da homogeneidade dos dados em um nó da árvore. Quanto menor a impureza, um nó tende a conter principalmente amostras de uma única classe, enquanto um nó impuro contém amostras de várias classes diferentes. Foi gerada a matriz de confusão de cada um dos modelos para análise da acurácia da classificação obtida na base de teste.

Finalmente, foram elaborados dois *heatmaps* (mapas de calor), um para o setor público e outro para o setor privado, apresentando a cobertura de registro de partos por UF, segundo as características hospitalares identificadas como preditoras nas etapas anteriores.

Em todas as análises, foi utilizado o programa estatístico R em sua interface RStudio (https://rstudio.com/). Para o tratamento dos dados, a criação do modelo da árvore de decisão e sua visualização, foram utilizados, respectivamente, os pacotes *caret*, *rpart* e *rpart.plott*, e, para o tratamento dos dados e a criação do modelo de floresta aleatória, foram utilizados os pacotes *caret* e *randomForest*.

Este estudo utilizou apenas bases de acesso público não identificadas, estando isento de apreciação ética.

## Resultados

Foram identificadas 26.108.417 DN em mulheres de 10 a 49 anos no período de 2012-2020. Após a exclusão de DN não hospitalares, DN em hospitais com menos de 100 nascidos vivos/ano, DN em hospitais sem convênio SUS e DN com CNES não preenchido ou inválido, 19.844.913 DN (76%), registradas em 2.824 hospitais, foram incluídas na análise. No SIH/SUS, foram identificadas 17.506.933 AIH com registro de procedimentos de parto em mulheres de 10 a 49 anos no período de 2012-2020. Após a exclusão das AIH identificadas em hospitais com menos de 100 nascidos vivos/ano e aquelas com CNES não preenchido ou inválido, 17.011.418 (97,2%) internações, ocorridas em 2.749 hospitais, foram incluídas na análise. Após a identificação de 19.573.019 DN e 17.011.418 AIH com número de CNES preenchido, existente e igual nas duas bases, foi estimada cobertura de registro de partos no SIH/SUS de 86,9%, com redução para 80,6% após a exclusão dos hospitais que apresentavam cobertura de registro de parto superior a 100% (Figura 1).

**Figura 2:**
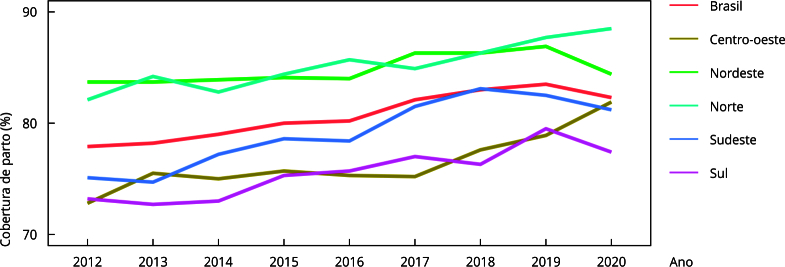
Série temporal da cobertura de partos no Sistema de Informação Hospitalar do Sistema Único de Saúde (SIH/SUS), por macrorregião do país, após exclusão dos hospitais com cobertura superior a 100%. Brasil, 2012-2020.

A análise por UF mostra que a proporção de falhas de registro do número de CNES no SINASC variou nos diversos estados brasileiros, sendo as maiores proporções observadas nos estados de Goiás (15,1%) e Roraima (12,1%). As falhas de registro no SIH/SUS foram pouco frequentes em todas as UF, mas as inconsistências entre o registro do CNES no SINASC e no SIH/SUS variaram, com proporções mais elevadas nos estados do Mato Grosso (4,9%), Paraná (4,9%) e Goiás (4,8%). Essas inconsistências resultaram, em alguns casos, em hospitais com valores de cobertura superior a 100%, sendo excluídos da análise. A cobertura de registro de partos, após a exclusão de hospitais com cobertura superior a 100%, variou de 72,2% a 91,1% no país, sendo mais baixa em estados das regiões Sul e Sudeste e mais elevada em estados das regiões Centro-oeste, Nordeste e Norte (Tabela 1). A tendência de aumento da cobertura de registro no período analisado foi significativa em todas as macrorregiões (Figura 2), com forte correlação positiva e variação de 0,8 a 0,97.

**Tabela 1:** Avaliação da cobertura de partos por Unidade Federativa (UF) e macrorregião no Sistema de Informação Hospitalar do Sistema Único de Saúde (SIH/SUS). Brasil, 2012-2020.

UF	SINASC		SIH/SUS		Pareamento SINASC-SIH/SUS		
	DN de mulheres de 10-49 anos com nascido vivo em hospital	DN com CNES válido em estabelecimentos com > 100 nascidos vivos/ano e com leito obstétrico conveniado ao SUS		AIH para parto, em mulheres de 10 a 49 anos, com CNES válido	AIH de internação para parto com CNES válido em hospitais com mais de 100 nascidos vivos/ano		DN/AIH com CNES válido e igual nas duas bases			Cobertura registro de partos no SIH/SUS *	Cobertura ≤ 100% **
	n	n	% ***	n	n	%	DN	% ^#^	AIH	%	%
							n		n		
Norte	2.667.418	2.313.151	86,7	2.109.163	2.051.489	97,3	2.281.825	98,6	2.051.489	89,9	85,0
Acre	138.554	134.193	96,9	116.009	112.436	96,9	133.755	99,7	112.436	84,1	80,8
Amazonas	654.875	568.336	86,8	487.370	484.150	99,3	555.795	97,8	484.150	87,1	83,1
Amapá	132.117	127.137	96,2	116.328	114.238	98,2	126.710	99,7	114.238	90,2	83,7
Pará	1.187.250	1.048.922	88,3	972.788	941.975	96,8	1.032.285	98,4	941.975	91,3	86,8
Rondônia	239.850	160.809	67,0	157.551	144.239	91,6	159.640	99,3	144.239	90,4	78,4
Roraima	96.636	88.164	91,2	85.993	84.242	98,0	88.164	100,0	84.242	95,6	88,2
Tocantins	218.136	185.590	85,1	173.124	170.209	98,3	185.476	99,9	170.209	91,8	88,6
Nordeste	7.212.822	6.024.517	83,5	5.674.640	5.455.764	96,1	5.962.366	99,0	5.455.764	91,5	84,8
Alagoas	454.896	406.155	89,3	379.733	371.599	97,9	404.428	99,6	371.599	91,9	87,9
Bahia	1.755.337	1.448.661	82,5	1.401.864	1.315.572	93,8	1.431.391	98,8	1.315.572	91,9	85,7
Ceará	1.142.770	989.679	86,6	907.759	887.451	97,8	985.440	99,6	887.451	90,1	84,8
Maranhão	990.990	867.772	87,6	761.154	735.442	96,6	861.369	99,3	735.442	85,4	80,7
Paraíba	514.765	440.165	85,5	373.854	366.247	98,0	427.536	97,1	366.247	85,7	76,6
Pernamabuco	1.229.568	951.547	77,4	928.784	901.496	97,1	935.232	98,3	901.496	96,4	87,6
Piauí	414.681	344.599	83,1	359.685	340.159	94,6	342.914	99,5	340.159	99,2	88,2
Rio Grande Norte	410.464	340.480	83,0	332.318	320.166	96,3	338.826	99,5	320.166	94,5	88,6
Sergipe	299.351	235.459	78,7	229.489	217.632	94,8	235.230	99,9	217.632	92,5	85,9
Centro-oeste	2.128.946	1.461.000	68,6	1.328.527	1.293.126	97,3	1.410.791	96,6	1.293.126	91,7	76,3
Distrito Federal	386.942	261.473	67,6	357.482	354.224	99,1	254.397	97,3	354.224	139,2	91,1
Goiás	864.230	476.104	55,1	387.752	369.979	95,4	453.171	95,2	369.979	81,6	74,3
Mato Grosso Sul	384.198	331.426	86,3	267.285	259.704	97,2	330.614	99,8	259.704	78,6	74,5
Mato Grosso	493.576	391.997	79,4	316.008	309.219	97,9	372.609	95,1	309.219	83,0	79,2
Sudeste	10.184.958	7.266.539	71,3	6.178.166	6.050.513	97,9	7.205.628	99,2	6.050.513	84,0	79,1
Espírito Santo	491.256	377.214	76,8	315.424	311.287	98,7	372.925	98,9	311.287	83,5	79,9
Minas Gerais	2.322.517	1.858.137	80,0	1.574.751	1.540.311	97,8	1.847.709	99,4	1.540.311	83,4	79,4
Rio de Janeiro	1.972.066	1.342.870	68,1	1.058.375	1.009.304	95,4	1.320.332	98,3	1.009.304	76,4	72,2
São Paulo	5.399.119	3.688.318	68,3	3.229.616	3.189.611	98,8	3.664.662	99,4	3.189.611	87,0	81,7
Sul	3.494.563	2.779.706	79,5	2.216.430	2.160.526	97,5	2.712.409	97,6	2.160.526	79,7	75,6
Paraná	1.385.420	1.094.476	79,0	864.282	840.276	97,2	1.041.227	95,1	840.276	80,7	75,7
Rio Grande do Sul	1.255.558	1.008.649	80,3	788.404	767.109	97,3	1.002.009	99,3	767.109	76,6	73,0
Santa Catarina	853.585	676.581	79,3	563.744	553.141	98,1	669.173	98,9	553.141	82,7	79,2
Brasil	25.688.707	19.844.913	77,3	17.506.926	17.011.418	97,2	19.573.019	98,6	17.011.418	86,9	80,6

AIH: Autorização de Internação Hospitalar; CNES: Cadastro Nacional de Estabelecimentos de Saúde; DN: Declaração de Nascido Vivo; SINASC: Sistema de Informações sobre Nascidos Vivos.

* Cobertura de registro de parto no SIH/SUS = número de AIH para parto dividido pelo número de DN multiplicado por 100;

** Cobertura ≤ 100%: cobertura de registro de parto no SIH/SUS após exclusão de hospitais com cobertura > 100%;

*** Proporção de DN em hospitais com mais de 100 nascidos vivos/ano e com leito obstétrico conveniado ao SUS e que apresentavam CNES preenchido e válido;

^#^ Proporção de DN em hospitais com mais de 100 nascidos vivos/ano, com leito obstétrico SUS e com número de CNES preenchido e válido e identificado no SIH/SUS.

Na análise das características do estabelecimento associadas à cobertura de registro de partos no SIH/SUS, verificamos que os maiores valores de cobertura foram observados em estabelecimentos públicos (federal 88,8%, estadual 87,2%, municipal 86,2%, privado 75,2%), naqueles com maior número de partos (até um parto/dia 71,9%, 2 a 6 partos/dia 77,7%, ≥ 7 partos/dia 84,7%), com maior proporção de leito obstétrico SUS (proporção ≤ 75% cobertura 67,6%, proporção > 75% cobertura 84,6%), com menor proporção de cesariana (proporção ≤ 38,9% cobertura de 89,7%, proporção maior que 71,7% cobertura de 63%) e com atividade de ensino (universitária 86,6%, outras modalidades de ensino 85,4%, sem atividade de ensino 78,7%), não sendo observadas diferenças segundo complexidade do hospital (alta 80,2%, média 80,9%, baixa 80,4%).

**Figura 3:**
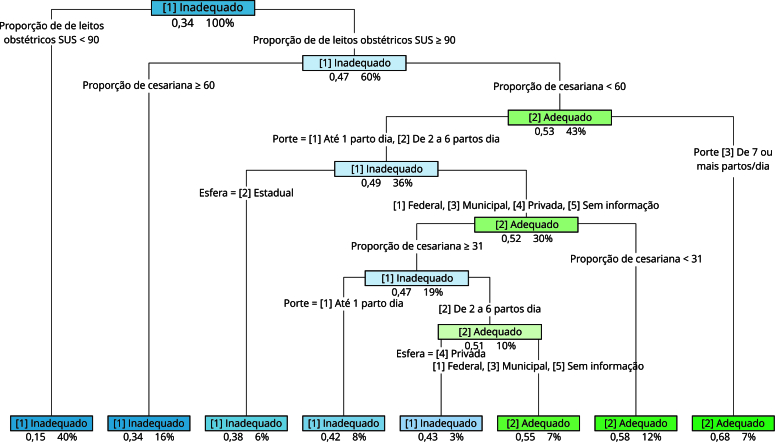
Árvore de decisão para cobertura de registro de partos no Sistema de Informação Hospitalar do Sistema Único de Saúde (SIH/SUS). Brasil, 2012-2020.

**Figura 4:**
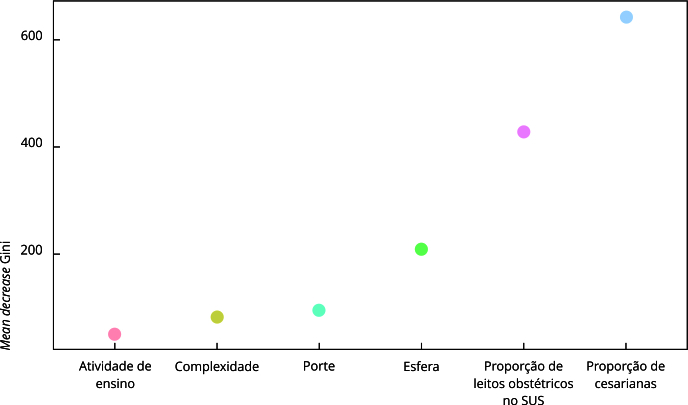
Floresta aleatória para cobertura de registro de partos no Sistema de Informação Hospitalar do Sistema Único de Saúde (SIH/SUS). Brasil, 2012-2020.

Na árvore de decisão, que considerou como parâmetro de adequação a cobertura de registro igual ou superior a 90%, as variáveis “proporção de leito obstétrico SUS” e “proporção de cesariana” apareceram nos primeiros nós da árvore. Foram classificados com maior probabilidade de cobertura adequada: hospitais de grande porte (≥ 7 partos dia), com proporção de leito obstétrico SUS ≥ 90% e proporção de cesariana menor que 60%, independentemente da esfera hospitalar; hospitais com alta proporção de leito obstétrico SUS (≥ 90%), baixa proporção de cesariana (< 31%), porte baixo ou médio (até 1 parto por dia e entre 2 a 6 partos, respectivamente), das esferas municipal, federal e privada ou sem informação; e hospitais municipais, federais ou sem informação, de médio porte (2 a 6 partos/dia), com alta proporção de leito obstétrico SUS (≥ 90%) e média proporção de cesariana (≥ 31% e < 60%) (Figura 3). No modelo de floresta aleatória, o resultado da métrica de *mean decrease* Gini elencou as variáveis “proporção de cesariana”, “proporção de leito obstétrico SUS”, “esfera administrativa” e “porte” com os valores mais altos (Figura 4). Os dois modelos apresentaram acurácias semelhantes (árvore de decisão 72,5%, floresta aleatória 70,7%), porém, com performances um pouco distintas com relação às predições dos estabelecimentos com cobertura adequada ou não. A árvore de decisão apresentou maior valor preditivo positivo (84% *vs*. 75,5%) e especificidade (59,7% *vs*. 47%), enquanto a floresta aleatória apresentou maior sensibilidade (85,9% *vs*. 74,6%) e valor preditivo negativo (45,4% *vs*. 63,5%).

**Figura 5:**
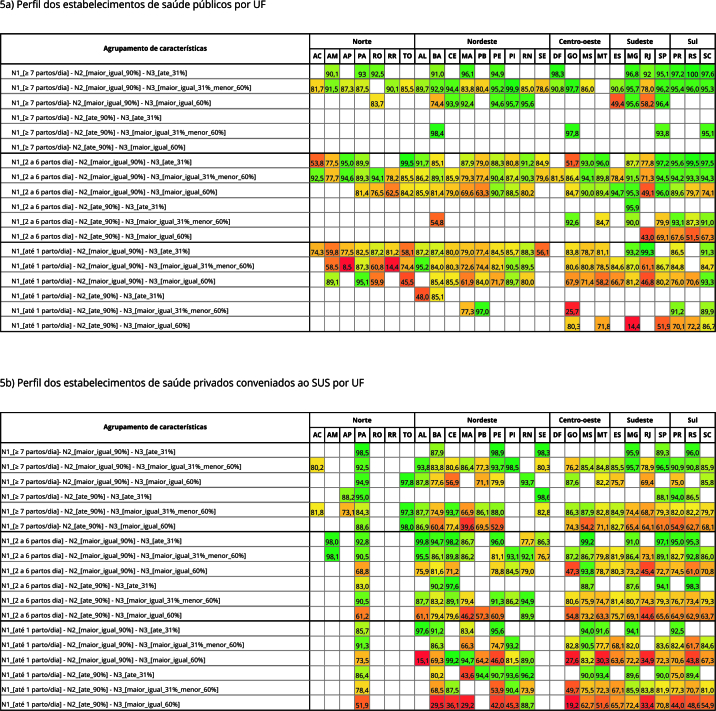
*Heatmap* da cobertura de partos (%) no Sistema de Informação Hospitalar do Sistema Único de Saúde (SIH/SUS) por Unidade Federativa (UF) segundo porte do hospital, proporção de leitos obstétrico SUS e proporção de cesarianas. Brasil, 2012-2020.

N1: porte; N2: proporção de leito SUS; N3: proporção de cesariana.

Na Figura 5, a cobertura de registro de partos por UF é apresentada segundo os principais fatores preditores identificados (porte do hospital, proporção de leitos obstétricos SUS e proporção de cesarianas) em hospitais públicos e privados conveniados ao SUS. Em ambos os hospitais, coberturas mais elevadas foram observadas nos estabelecimentos grandes (≥ 7 partos/dia), com proporção de leitos SUS ≥ 90% e proporção de cesariana < 31% e menores coberturas em hospitais de pequeno porte (até 1 parto/dia), com proporção de leito SUS < 90% e proporção de cesariana elevada.

## Discussão

Os resultados encontrados neste estudo demonstram uma cobertura de registros de parto no SIH/SUS de 80,6% no país, com aumento no período analisado, e com maior cobertura nas macrorregiões Norte e Nordeste. Entre os fatores preditivos, a elevada proporção de nascimentos por cesariana foi o principal fator preditivo da menor cobertura de registro de partos, enquanto a proporção de leitos conveniados ao SUS, a esfera administrativa e o porte do hospital foram os principais fatores preditivos da maior cobertura.

A cobertura encontrada, após a exclusão dos hospitais com cobertura superior a 100%, ficou abaixo da estimada por Machado et al. ^15^ em estudo realizado com as bases do SINASC e SIH/SUS do ano 2009, de 87,5%, com variação de 78,3% na Região Centro-oeste a 90,3% na Região Nordeste. A análise sem a exclusão desses hospitais estimou valores semelhantes à cobertura nacional (86,9%) relatada pelos mesmos autores ^15^, porém, com diferenças nas coberturas regionais, que variaram de 79,7% na Região Sul a 91,7% na Região Centro-oeste.

A elevada cobertura de registro de parto no SIH/SUS observada em todas as regiões e o aumento no período analisado indicam uma estabilidade no registro de partos do SIH/SUS. Entretanto, falhas no registro do CNES afetaram as estimativas encontradas. Foram observadas falhas principalmente no registro do SINASC, com número de CNES não preenchido ou inexistente, que variaram em frequência nas diferentes UF, e podem ser objeto de melhoria na gestão local. Um outro problema identificado foi o uso de número de CNES diferente no SINASC e no SIH/SUS, resultando na exclusão de DN com CNES sem correspondência no SIH/SUS, bem como valores de cobertura superiores a 100%. Uma situação identificada foi a existência de serviço de maternidade em hospital geral, sendo utilizado o CNES do serviço de maternidade no SINASC e o CNES do hospital geral no SIH/SUS. Outra situação identificada foi a mudança do CNES durante o período analisado, com a atualização do número no SIH/SUS e a manutenção do número antigo no SINASC. Essas situações específicas devem ser investigadas em cada contexto.

No estudo realizado por Correa et al. ^12^ no Município do Rio de Janeiro, analisando a cobertura do SIH/SUS segundo local de residência da mulher, também foram observados valores improváveis, superiores a 100% ou muito próximo a zero, o que os autores atribuíram a erros no registro da residência da mulher nos dois sistemas, problema já referido em estudo anterior no Município do Rio de Janeiro ^16^. Os resultados encontrados neste estudo e no estudo de Correa et al. ^12^ reforçam a importância do registro correto das informações em todos os formulários utilizados pela instituição que alimentarão os diversos sistemas de informação nacionais.

Quanto aos fatores preditivos identificados, menor registro de cesarianas já havia sido reportado por Bittencourt et al. ^16^ em estudo de avaliação da cobertura do SIH/SUS no Rio de Janeiro. Nos anos 1990, o Governo Federal publicou uma série de medidas para conter o aumento de cesarianas no país; entre elas, várias portarias que definiam um limite máximo de cesarianas por UF e hospitais ^21^ ^,^ ^22^ ^,^ ^23^ ^,^ ^24^ ^,^ ^25^ ^,^ ^26^. Essas portarias só foram revogadas em 2017 ^27^ e são a explicação mais provável para a menor cobertura de registro em estabelecimentos com maior proporção de nascimentos por cesariana, já que o pagamento de internações com realização de cesariana era rejeitado quando o limite máximo de cesarianas da instituição hospitalar já tivesse sido alcançado.

Deve-se ressaltar que não foram observadas diferenças na cobertura de registro de partos segundo o nível de complexidade do hospital. Sendo a proporção de cesarianas um fator associado ao menor registro de partos, seria de se esperar um menor registro de partos em hospitais de maior complexidade, já que esses deveriam ter uma proporção maior de nascimentos por cesariana por prestar assistência a gestantes de maior risco. Esse resultado, no entanto, é coerente com estudos realizados no Brasil, que mostram que a cesariana é um procedimento realizado com frequência no país, independentemente do risco gestacional materno ^28^.

A maior cobertura em hospitais com maior proporção de leitos conveniados ao SUS é esperada, já que, em hospitais com baixa proporção de leitos SUS, muitos nascimentos registrados no SINASC não estão registrados no SIH/SUS, por não terem financiamento público. Cabe ressaltar que essa característica foi importante tanto em hospitais privados como em hospitais públicos, indicando que, nesses últimos, nem todos os leitos são financiados pelo SUS, embora o hospital seja público. Hospitais de servidores municipais e estaduais são exemplos de estabelecimentos públicos em que a proporção de leitos do SUS foi inferior a 100%. A inclusão da fonte de financiamento do parto na DN permitiria uma análise mais adequada da cobertura de registro de parto no SIH/SUS nesses hospitais. Já a maior cobertura em hospitais com maior número de partos sugere uma melhor organização dos registros do SIH/SUS em estabelecimentos nos quais a internação para partos é mais frequente. Maior cobertura do registro de internações em hospitais de maior porte também foi relatado por Machado et al. ^15^.

Os modelos de predição apresentaram acurácia semelhante, diferindo nas medidas específicas, com maior sensibilidade e valor preditivo negativo na floresta aleatória. Entretanto, apesar dessas diferenças, as variáveis identificadas como mais importantes no modelo da floresta aleatória estavam contidas na árvore de decisão, e os resultados da árvore podem ser de mais fácil implementação pelos gestores, uma vez que geram pontos de corte para as variáveis, permitindo a visualização de “regras de classificação” e respectivos valores. Por exemplo, na árvore apresentada, hospitais com proporção de leitos SUS < 90% e hospitais com proporção de leitos SUS ≥ 90% e proporção de cesarianas ≥ 60% apresentaram a maior probabilidade de cobertura inadequada de registro de parto, representando 56% da base, devendo ser o foco prioritário de estratégias de melhoria.

Este estudo tem algumas limitações. Para o estudo da morbidade materna, o evento de interesse é a internação obstétrica e não apenas a internação para o parto, mas como não existem outros sistemas de informação que contenham esse dado para comparação, a cobertura de partos tem sido utilizada como *proxy*, sendo o SINASC o padrão de referência. Portanto, os resultados encontrados referem-se apenas a partos. Entretanto, estudo recente que realizou a validação do SIH/SUS para análise de internações obstétricas - incluindo internações para a assistência ao parto, ao aborto e a intercorrências na gestação e no puerpério - e utilizando dados de coleta de prontuário como padrão de referência, encontrou-se cobertura global de registro de internações obstétricas de 95% ^29^. Nesse estudo, apenas hospitais com mais de 2.750 partos por ano foram incluídos, sendo a cobertura mais elevada coerente com dados deste estudo, que mostrou maior cobertura de registro em hospitais de maior porte. Uma outra limitação é a utilização do total de internações por parto no SIH/SUS, com a inclusão apenas de partos de nascidos vivos no SINASC. Entretanto, como a taxa de natimortos no Brasil foi de aproximadamente 10 por mil (1% dos partos) no período analisado ^30^, é pouco provável que a inclusão de partos de natimortos tenha afetado as estimativas de cobertura apresentadas. No SINASC, foram incluídos todos os registros de nascimento, o que inclui gestações múltiplas, enquanto, no SIH/SUS, não é possível identificar as gestações gemelares, podendo as mesmas resultarem em uma ou mais AIH (por exemplo, no caso de um nascimento por via vaginal e outro por cesariana). Como a proporção de gestações gemelares no país no período analisado foi de 2%, com variação de 1,5% a 2,5% nas UF ^30^, pode-se esperar um efeito pequeno na estimativa de cobertura de registro de partos no SIH/SUS em decorrência de diferenças de registro nessas gestações. Por fim, foram excluídos hospitais com menos de 100 nascidos vivos/ano. Embora menos de 3% dos nascimentos vivos no país ocorram nesses hospitais ^31^, é possível que hospitais menores apresentem qualidade de registro diferente da apresentada neste artigo.

## Conclusão

O SIH/SUS apresentou elevada cobertura para registro de partos no país, sendo identificadas falhas que podem ser corrigidas visando melhor desempenho do sistema. Especial atenção deve ser dada aos hospitais com menor número de partos, bem como para aqueles com elevada proporção de cesariana.
